# Blackgrass (*Alopecurus myosuroides* Huds.) Multiple Resistance to ACCase- and ALS-Inhibitors and Its Competition with Winter Wheat

**DOI:** 10.3390/genes16101169

**Published:** 2025-10-03

**Authors:** Aristeidis P. Papapanagiotou, Ioannis Vasilakoglou, Maria V. Alvanou, Ioannis A. Giantsis, Panagiotis Madesis, Ilias G. Eleftherohorinos

**Affiliations:** 1 Department of Agriculture, University of Western Macedonia, 53100 Florina, Greece; 2 Department of Agriculture-Agrotechnology, University of Thessaly, 41500 Larissa, Greece; vasilakoglou@uth.gr (I.V.); eleftero@agro.auth.gr (I.G.E.); 3 School of Agriculture, Aristotle University of Thessaloniki, 54124 Thessaloniki, Greece; malvano@agro.auth.gr; 4 Department of Agriculture Crop Production and Rural Environment, University of Thessaly, 38446 Nea Ionia, Greece; pmadesis@uth.gr; 5 Department of Field Crops and Ecology, Aristotle University of Thessaloniki, 54006 Thessaloniki, Greece

**Keywords:** metabolism, piperonyl butoxide (PBO), non-target-site resistance (NTSR), target-site resistance (TSR)

## Abstract

**Background/Objectives:** The herbicide resistance of blackgrass (*Alopecurus myosuroides* Huds.) is one of the most serious problems in the winter cereal monoculture in Europe. Recently, Greek farmers expressed complaints of reduced susceptibility of this weed to winter wheat herbicides. Keeping this in mind, this study focused on the investigation of blackgrass resistance to herbicides at both phenotypic and molecular levels. **Methods**: Whole-plant rate-response pot assays were conducted to study the possible evolution of resistance (cross- or multiple-resistance) in a blackgrass population to ACCase- and ALS-inhibiting herbicides. Analysis of the *ACCase* gene sequence, herbicide metabolism study and competition with winter wheat studies were also conducted. **Results**: High levels of cross-resistance mainly to the ACCase post-emergence clodinafop-propargyl, medium to fenoxaprop-P-ethyl, cycloxydim, pinoxaden, as well as lower levels of resistance to ALS-inhibitors (mesosulfuron-methyl + iodosulfuron-methyl-sodium and pyroxsulam) were confirmed. In addition, the pre-emergence soil-applied herbicides chlorotoluron + diflufenican and prosulfocarb provided excellent control of the S and R blackgrass populations. The analysis of the ACCase gene sequence revealed a point mutation at position 1781, resulting in an amino acid substitution from isoleucine (Ile) to leucine (Leu). Furthermore, the combined application of the herbicides with piperonyl butoxide (PBO, applied 2 h before herbicide application) indicated that there was herbicide metabolism, which may be mediated by cytochrome P450. The R blackgrass population, when grown in competitive interaction with winter wheat, produced more tillers and aboveground fresh weight compared to the S population and caused greater reduction in winter wheat. **Conclusions**: The results suggest that a blackgrass population has developed multiple resistance to ACCase- and ALS-inhibiting herbicides, due to *ACCase* gene mutation and herbicide metabolism. No fitness cost and no compromised competitive ability associated with the blackgrass resistance were observed.

## 1. Introduction

Blackgrass (*Alopecurus myosuroides* Huds.) is a noxious annual, diploid, allogamοus grass weed, propagated solely by seeds. It is a temperate species with an optimum temperature for seed germination at 15–25 °C. The species occurs throughout much of Europe but is a troublesome weed in the low-lying arable areas of England, France, Germany, Belgium, Spain, Switzerland, The Netherlands, Poland, and Greece [1]. Blackgrass is highly adapted to small grain cereal crops and thrives in heavy clay or silt-loamy, waterlogged soils, but is not well-suited to lighter sandy or gravelly ones.

Blackgrass is mainly cross-pollinated, as it possesses a self-incompatibility system, which is characterized by a high reproductive capacity, allowing rapid increase in weed populations in winter cereal land. Dense infestations of blackgrass in cereal fields can result in substantial yield losses, reaching 45% [2]. It is worth mentioning that a density of 12 blackgrass plants per m^2^ can lead to a 5% yield reduction in small grain winter cereals [3], and this is used to indicate the threshold where chemical control is justified. Competition ability varies depending on location and various factors such as soil type, nitrogen level, and weather conditions (especially rainfall) [4]. Moreover, the competitiveness of blackgrass depends greatly on the vigor of the crop, which influences the weed tillering and lodging ability in infested winter cereal fields, resulting in slower harvesting and poor grain quality.

Extensive application of selective post-emergence herbicides, mainly acetyl-coenzyme A carboxylase (ACCase)- and acetolactate synthase (ALS)-inhibitors is the most common means of blackgrass control in small grain cereals. Both modes of action are characterized as high risk in imposing strong selection pressure on weed populations and resulting in the rapid evolution of herbicide target-site resistance [3]. Over time, the repeated use of grass-inhibiting herbicides has resulted in the field-selection of numerous blackgrass populations, which is now the most important herbicide-resistant weed in Europe, as it occurs in at least 14 countries [5]. Initially, herbicide resistance evolved in substituted ureas [1], followed by resistance to ACCase-inhibiting herbicides [6]. At present, diversified resistance-endowing mechanisms such as target-site point mutations and enhanced herbicide detoxification [7] among blackgrass populations are responsible for compromised chemical control by herbicides belonging to seven different herbicide modes of action [8]. Blackgrass field-evolved herbicide resistance is now very widespread across the geographical range at most major cereal-producing areas of Europe, where it affects most arable farms and renders the ACCase [9] and ALS inhibitors [10] ineffective. Moreover, blackgrass populations exhibit multiple resistance to both modes of action [11] and resistance to multiple herbicide classes [12].

Resistance to herbicides is conferred by two different mechanisms: target-site resistance (TSR) and non-target-site resistance (NTSR) [13]. The considerable variation in preexisting TSR haplotypes due to standing genetic variation in blackgrass is responsible for the rapid selection of herbicide-resistant populations infesting arable fields [9]. ACCase target-site resistance is conferred by coding sequence mutations that alter the expression of the herbicide target protein or give rise to a functional enzyme with reduced target-binding affinity for herbicides, or overexpression (amplification) of the specific genes encoding the proteins targeted by herbicides [9]). The ACCase target-site mechanism is very common among field-evolved blackgrass populations, and this is confirmed by the seven demonstrated point mutations in the gene-encoding resistance to ACCase inhibitors [14]. However, the most frequent ACCase target-site resistance in blackgrass populations is conferred by mutations at the Ile-1781 codon [14,15]. ALS target-site mediated herbicide resistance most commonly endowed by point mutations at Pro-197 and Trp-574 codon positions is alarmingly increasing among blackgrass populations, impacts the efficacy and threatens the sustainable use of ALS-inhibiting herbicides [16]. The outcrossing nature of blackgrass enables the combination and accumulation of different resistance genes in individual plants, conferring multiple resistance to both ACCase- and ALS-inhibitor herbicides.

The NTSR is endowed by changes in the rates of herbicide absorption, translocation, degradation, or sequestration, thereby neutralizing the herbicides or reducing herbicide access to their site of action. Enhanced metabolism-based resistance involves the increased expression of Cytochrome P450 monoxygenases, glutathione S-transferases (GSTs), UDP-glycosyltransferases (UGTs), and ATP-binding cassette (ABC) transporters [8]. It is considered a generalist herbicide resistance mechanism that confers unpredictable resistance patterns to chemically dissimilar pre- and post-emergence selective herbicides with various modes of action, including even those not yet marketed [17]. NTSR detoxifying mechanisms are predominant in numerous ACCase inhibitor-resistant blackgrass populations [18], which often co-exist with TSR in many field-evolved blackgrass populations [19]. As reported by Kersten et al. [9], the ratio of TSR to NTSR among field-selected blackgrass populations displays a significant variation, with some populations having either a TSR-conferring mechanism or other populations in which both TSR and NTSR mechanisms co-exist.

Due to farmers’ complaints regarding reduced efficacy of ACCase inhibitors against a blackgrass population in a small grain cereal monoculture field, the objectives of this study were as follows: i. to test one putative resistant (R) blackgrass population for developing resistance to ACCase (clodinafop propargyl, fenoxaprop-P-ethyl, pinoxaden, cycloxydim) and ALS (mesosulfuron-methyl + iodosulfuron-methyl-sodium, pyroxsulam) inhibitors, ii. to evaluate the efficacy of pre-emergence herbicides for the management of the R blackgrass population, iii. to elucidate the mechanism of resistance involved, and iv. to assess the competitive ability of the R blackgrass and compare it with that of a susceptible (S) blackgrass population when grown in competition with winter wheat.

## 2. Materials and Methods

### 2.1. Seed Material

Blackgrass seeds were collected from one field located in the prefecture of Grevena, northwestern Greece (40.206196 N, 21.410579 E), where small grain cereal monoculture had been practiced for at least 15 years. Treatments mainly with ACCase- and occasionally with ALS-inhibitors had been employed to manage grass weed infestations in that crop field effectively. The application of the aryloxyphenoxypropionate herbicide clodinafop-propargyl in the spring of 2020 failed to provide satisfactory blackgrass control. Thus, mature weed seeds were collected during seed shedding by hand from a large number of plants (from different patches) before winter wheat was harvested and bulked. The seeds were pooled together and regarded as a putative resistant (R) population. Moreover, mature seeds were collected from the field margins of a close winter wheat field (40.206688 N, 21.410122 E) infested with a blackgrass population against which clodinafop-propargyl provided excellent control, and was therefore considered as a susceptible (S) population. The seeds of both blackgrass populations were initially transported to the laboratory. They were subsequently cleaned, air-dried, and stored in paper bags in a chamber maintained at 5–7 °C, until use in subsequent pre-emergence and post-emergence whole-plant rate-response pot experiments.

### 2.2. Whole-Plant Rate-Response to Post-Emergence Herbicides

**Figure 1:**
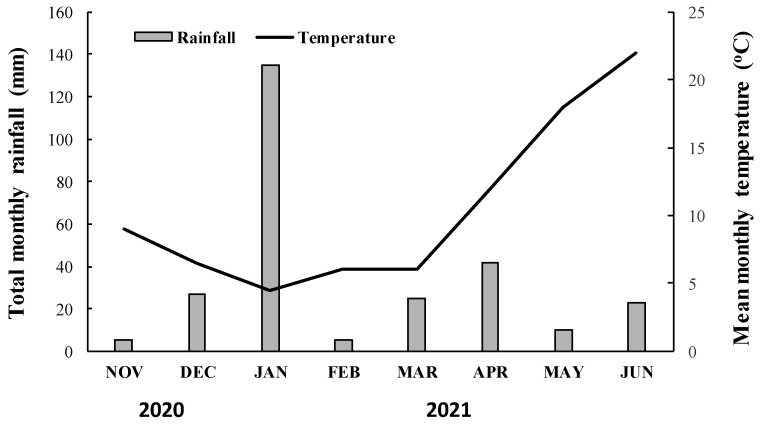
Total monthly rainfall (mm) and mean monthly temperature (°C) recorded during the 2020–2021 growing season near the experimental area.

The putative resistant (R) and the susceptible (S) blackgrass populations were studied in two whole-plant rate-response assays repeated over time. The experiments were conducted at the farm of the University of Western Macedonia (Florina), during the 2020/21 growing season. The experiments were established in 10 × 10 × 10 cm^3^ plastic pots filled with a mixture of clay loam soil–peat–sand (1:1:1; *v*/*v*/*v*). Each pot was seeded with approximately 15 blackgrass seeds and carefully covered with the soil mixture (0.5 cm depth). When blackgrass plants reached the two-leaf stage, they were carefully thinned to six evenly sized plants per pot. Herbicide treatments were performed when blackgrass plants of the R and S populations reached the four- to five-leaf stage (BBCH code 14–15) (1–2 tillers) [20]. To confirm possible selection of ACCase inhibitor resistance in the suspected blackgrass population, herbicides were sprayed at rates corresponding to 1.0, 2.0, 4.0, and 8.0 times their recommended label field rate. Blackgrass plants of the putative R population were sprayed with the ACCase-inhibiting herbicides clodinafop-propargyl (Sword^®^ 240 EC, K&N Efthymiadis S.A., Thessaloniki, Greece) (at 40.8, 81.6, 163.2, 326.4 g ai ha^−1^), fenoxaprop-P-ethyl (Fenova^®^ Super 6.9EW, FMC Hellas, Athens, Greece) (at 82.8, 165.6, 331.2, 662.4 g ai ha^−1^), pinoxaden (Axial^®^ 60 EC, Syngenta Hellas, Athens, Greece) (at 45, 90, 180, 360 g ai ha^−1^), cycloxydim (Focus 10 EC; BASF Hellas, Athens, Greece) (at 200, 400, 800, 1600 g ai ha^−1^) and the ALS-inhibitors mesosulfuron-methyl + iodosulfuron-methyl-sodium (Atlantis^®^ WG, Bayer Crop Science Hellas, Athens, Greece) (at 15 + 3, 30 + 6, 60 + 12, 120 + 24 g ai ha^−1^) and pyroxsulam (Senior 75 WG, Dow Elanco Hellas, Athens, Greece) (at 18.75, 37.5, 75, 150 g ai ha^−1^). All mesosulfuron-methyl + iodosulfuron-methyl-sodium and pyroxsulam treatments were applied in mixture with a non-ionic surfactant [Biopower (alkylethersulfate sodium salt 38.35% *w*/*w*) at 1.0 L ha^−1^]. In contrast, the S blackgrass population was treated with a one eighth (1/8×), a quarter (1/4×), half (1/2×), and the recommended label (1×) rate of the aforementioned ACCase- and ALS-inhibiting herbicides; clodinafop-propargyl (at 5.1, 10.2, 20.4, 40.8 g ai ha^−1^), fenoxaprop-P-ethyl (at 10.35, 20.7, 41.4, 82.8 g ai ha^−1^), pinoxaden (at 5.62, 11.25, 22.5, 45 g ai ha^−1^), cycloxydim (at 25, 50, 100, 200 g ai ha^−1^), mesosulfuron-methyl + iodosulfuron-methyl-sodium (at 1.87 + 0.37, 3.75 + 0.75, 7.5 + 1.5, 15 + 3 g ai ha^−1^), and pyroxsulam (at 2.34, 4.68, 9.37, 18.75 g ai ha^−1^). A 2.4 m wide boom field plot sprayer equipped with six 8002 flat-fan nozzles was used to apply all herbicide treatments. This propane-pressurized sprayer was calibrated to deliver 300 L ha^−1^ of spray solution at 280 kPa. Following the herbicide applications, pots were returned to a net-protected area outdoors and were watered as needed to maintain optimum growing conditions. The mean monthly temperature and total monthly rainfall data that prevailed throughout the study of both blackgrass populations near the experimental area are presented in Figure 1.

Each of the two identical whole-plant rate-response experiments were established in a completely randomized design with four replications for each treatment. Within each population, pot randomization was practiced weekly to ensure uniform growth conditions for all blackgrass plants. To assess the response of both PR and S blackgrass populations to the post-emergence ACCase- and ALS-inhibiting herbicides, shoots were cut at soil level four weeks after treatment (WAT) and the aboveground fresh weight of the surviving plants was recorded. Analysis of variance (ANOVA) was performed using the fresh weight data expressed as a percentage of the untreated control.

### 2.3. Rate-Response to Pre-Emergence Herbicides

One experiment was conducted and repeated at the farm of the University of Western Macedonia, Greece, during the fall of 2020 to early winter of 2021, to study the response of the R and S blackgrass populations to pre-emergence herbicides. The experiments were performed in 10 × 10 × 10 cm^3^ plastic pots filled with a mixture of clay loam soil–peat–sand (1:1:1; *v*/*v*/*v*), as previously described. Each pot was seeded with approximately 15 blackgrass seeds of the R or the S population and carefully covered with the soil mixture (1 cm depth). One week after seeding, treatments of pre-emergence herbicides were applied to the pot’s soil surface. The populations were treated with half the recommended (1/2×), recommended (1×) and twice (2×) the recommended field rate of two selective pre-emergence herbicides, registered for use in small cereal crops in Greece; prosulfocarb (Boxer 80 EC, Syngenta Hellas) was applied at 1800, 3600, 7200 g ai ha^−1^ and diflufenican + chlorotoluron (Constel F^®^ SC, Alpha Agricultural Supplies S.A., Athens, Greece) at 50 + 800, 100 + 1600, 200 + 3200 g ai ha^−1^, respectively. A nontreated control for both R and S blackgrass populations was also included in the experiments to allow comparison. The pre-emergence herbicides were applied by the hand-field, plot portable sprayer used in the rate-response experiments of post-emergence herbicides. After treatment, the residual herbicides were incorporated into the soil mixture by hand-watering the pots with a water volume corresponding to 10 mm of rainfall. All pots were placed outdoors in a net-protected area exposed to natural conditions prevailing during the normal winter growing season and were watered daily to keep the soil mixture moist. Pots were rearranged each week for all plants to achieve uniform growth and randomize any differences in environmental conditions. The experimental design was a completely randomized block with four replications per treatment. At six WAT, the emerged blackgrass plants were counted, and their fresh weight was evaluated. The fresh weight data were expressed as a percentage of the untreated control, and they were subjected to analysis of variance (ANOVA).

### 2.4. Amplification and Sequencing of ACCase Gene Fragments

The fragment harboring potential codon changes resulting in amino acid substitutions in the carboxyltransferase domain of the *ACCase* gene, implicated in ACCase-inhibitor resistance, was amplified and sequenced in plant samples of the R and S blackgrass populations as determined in preceding rate-response experiments. For *ACCase* gene amplification, four pots of each population were treated with the field label rate of clodinafop-propargyl, while an additional four pots of the S population were left untreated. For molecular analyses, 20 mg of leaf tissue from individual R plants that survived clodinafop-propargyl application and untreated S plants were harvested, weighed, and homogenized in liquid nitrogen. Isolation of genomic DNA was performed from each homogenate using the NucleoSpin Plant II Mini kit for DNA (Macherey-Nagel, Düren, Germany), according to the manufacturer’s protocol. The quantity and quality of the extracted DNA samples were evaluated in a Q5000 spectrophotometer (Quawell, Kowloon, Hong Kong). Fifty ng of isolated DNA were used for amplification of the *ACCase* gene using the primer pairs depicted in Table 1 [21,22]. In total, the four target-site point mutations Ile1781Leu, Ile2041Asn, Gly2078Asp, and Gly2096Ala were investigated.

The PCR reactions were carried out in an Eppendorf Mastercycler thermal cycler, in a total reaction volume of 20 μL. More specifically, in each reaction 0.6 pmol of each primer, 50 ng isolated DNA, 10 μL FastGene Taq 2X Ready Mix (Nippon Genetics, Europe, Dueren, Germany), and ultrapure water up to the final volume were included. PCR conditions consisted of one step for 5 min at 94 °C, followed by 36 cycles of 30 s at 94 °C, 30 s at optimal Tm for each primer pair (Table 1), and 40 s at 72 °C, followed by a final extension step of 72 °C for 5 min. The PCR products that were successfully amplified were purified using the NucleoSpin Gel and PCR Clean-up kit (Macherey-Nagel, Dueren, Germany) and were bidirectionally sequenced using the primer pair previously used for the PCR amplification in a 3730 automatic sequencer. Sequences were read, edited, and aligned in the software MEGA (Molecular Evolutionary Genetics Analysis) version X [25] in comparison with blackgrass *ACCase* gene (GenBank: AM408430.1) for the detection of the genotypes based on known ACCase point mutations such as Ile178l.

### 2.5. Metabolism Study

One pot experiment was carried out and repeated over time aiming to examine the possible presence of a non-target-site mediated resistance (NTSR) mechanism (enhanced metabolism due to elevated levels of herbicide-detoxifying enzymes like cytochrome P450 monoxygenases) in the R blackgrass population, contributing to the compromised efficacy of post-emergence ACCase- and ALS-inhibiting herbicides against it. The experiment investigated the potential of piperonyl butoxide (PBO) (Tokyo Chemical Industry Co., Ltd. (Tokyo, Japan)), a potent P450s inhibitor to reverse resistance in the field-selected R blackgrass population and enhance the herbicidal activity of ACCase- and/or ALS-inhibitors when applied prior to the selective post-emergence herbicide treatments. The experimental layout was a completely randomized design with four replicates for each treatment. A single rate of PBO (2 kg ai ha^−1^) was applied when blackgrass plants reached the BBCH code 14–15 (4–5 leaves, 1–2 tillers) [20]. The ACCase (clodinafop-propargyl, fenoxaprop-P-ethyl, pinoxaden, cycloxydim)- and the ALS (mesosulfuron-methyl + iodosulfuron-methyl-sodium, pyroxsulam)-inhibiting herbicides were applied in two rates, at the recommended maximum field label (1×) and twice the recommended (2×) rate against the R blackgrass population. The application of PBO was performed 2 h before the application of all post-emergence herbicides to allow its absorption and potential inhibition of the P450s activity. Herbicide applications were performed under similar conditions and with the same field plot sprayer used in the whole-plant, rate-response assays. An untreated control and a PBO treatment only were also included. The aboveground biomass of plants exposed to all treatments was harvested at four WAT. The mean aboveground green biomass for each treatment was determined. Fresh weight was subsequently expressed as a percentage of the PBO treatment that was equal to that of the untreated control.

### 2.6. Blackgrass Competition with Winter Wheat

**Figure 2:**
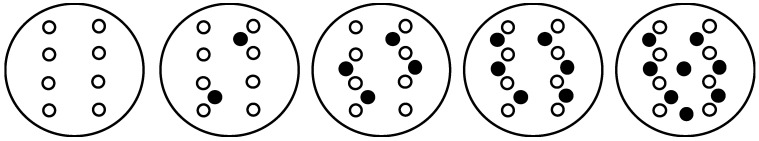
Schematic illustration of a target-neighborhood experiment with various crop/weed density patterns (8:0, 8:2, 8:4, 8:6, 8:8), designed to assess winter wheat response when grown alone and in competitive interaction with increasing density of the R (ACCase-herbicide-resistant) or the S (susceptible) blackgrass population [winter wheat plants = open circles vs. R or S blackgrass plants = black circles].

An inter-specific competition pot experiment was conducted and repeated from November 2020 to June 2021 to assess and compare the competitive abilities of the R and S blackgrass populations grown in competitive interaction with winter wheat (cv. Zanzibar) in a ‘target-neighborhood design’ for the crop. The first inter-specific competition pot experiment was established on 1 November and it was followed by a second identical experiment established one month later. Monthly temperature and precipitation data are presented in Figure 1. A template (shown in Figure 2) was used to ensure equal spacing between neighboring crops and weed plants. Plastic pots (20 × 20 × 15 cm^3^ were filled with the same soil mixture used in all previous experiments. Eight winter wheat hills were seeded in each pot, following the same procedure described in the whole-plant rate-response pot assays) in two rows, spaced 12 cm apart. Each row contained four hills, with two seeds per hill, placed 6 cm apart. When winter wheat plants reached the one-leaf stage, one plant was gently removed in each hill to allow one remaining wheat plant to grow per each hill, resulting in eight plants per pot (equivalent to 256,000 plants ha^−1^). Blackgrass seedlings at the one-leaf stage (same time of wheat plant thinning) were transplanted into the pots at five different densities: 0 (control with no weed competition), 2, 4, 6, and 8 blackgrass plants per pot (equivalent to 0, 63, 126, 189, and 252 plants m^−2^, respectively) (Figure 2). The pots were placed and kept outdoors in a natural outdoor environment, exposed to ambient conditions, and watered as needed. Fertilization was applied with 40 kg N ha^−1^ and 50 kg P ha^−1^ as ammonium phosphate before sowing, with an additional 25 kg N ha^−1^ in early April, complying with standard agronomic practices in the local small cereal production systems to support vigorous growth of both crop and weed. Throughout the experiment, any undesired weeds emerging from the soil mixture were hand-removed. Once the crop and weed plants reached the flag leaf sheath opening growth stage (BBCH code 47), two traits were recorded to assess plant growth: the aboveground fresh weight (a trait reflecting competitive ability) and the number of tillers produced by winter wheat and blackgrass plants. The experimental design was a randomized complete block design with four replications (pots) for both R and S blackgrass populations and weed density treatment.

### 2.7. Statistical Analyses

For the rate-response to post-emergence herbicides study, a combined over two experiments ANOVA was performed, separately for the R and S blackgrass populations, using a 6 (herbicides) × 4 (rates) factorial approach. For the rate-response to pre-emergence herbicides study, a 2 (blackgrass populations) × 2 (herbicides) × 3 (rates) factorial approach was used. Also, nonlinear regression analysis was performed using a four-parameter log-logistic equation [26] to estimate the herbicide dose required to reduce fresh weight by 50% (*GR*_50_):

y = c + (d − c)/{1 + exp[b(logx − log*GR*_50_)]}

where c and d represent lower and upper limits, b is the relative slope around *GR*_50_, x is the herbicide dose, and y is the percentage growth response.

For the metabolism study, a 6 (herbicides) × 4 (rates) factorial approach was used. The data obtained from the inter-specific experiments for winter wheat were also pooled together for ANOVA, following a factorial approach (2 blackgrass populations × 5 weed densities). Moreover, a factorial approach was used for blackgrass data (2 populations × 4 weed densities). Linear regression analysis was also applied to evaluate growth trends over weed density. In these regression equations, the blackgrass plants per pot (density) was the independent variable (x), and the plant parameters of either weed or winter wheat was the dependent variable (y).

The R software (version 4.4.1; R Foundation for Statistical Computing, Vienna, Austria) was used to estimate the parameters of the log-logistic curves [27], while the MSTAT-C program [28] was used to conduct the ANOVAs. Differences between means were compared at *p* < 0.05 using Fisher’s protected LSD (least significant difference) test.

## 3. Results

### 3.1. Whole-Plant Rate-Response to Post-Emergence Herbicides

**Figure 3:**
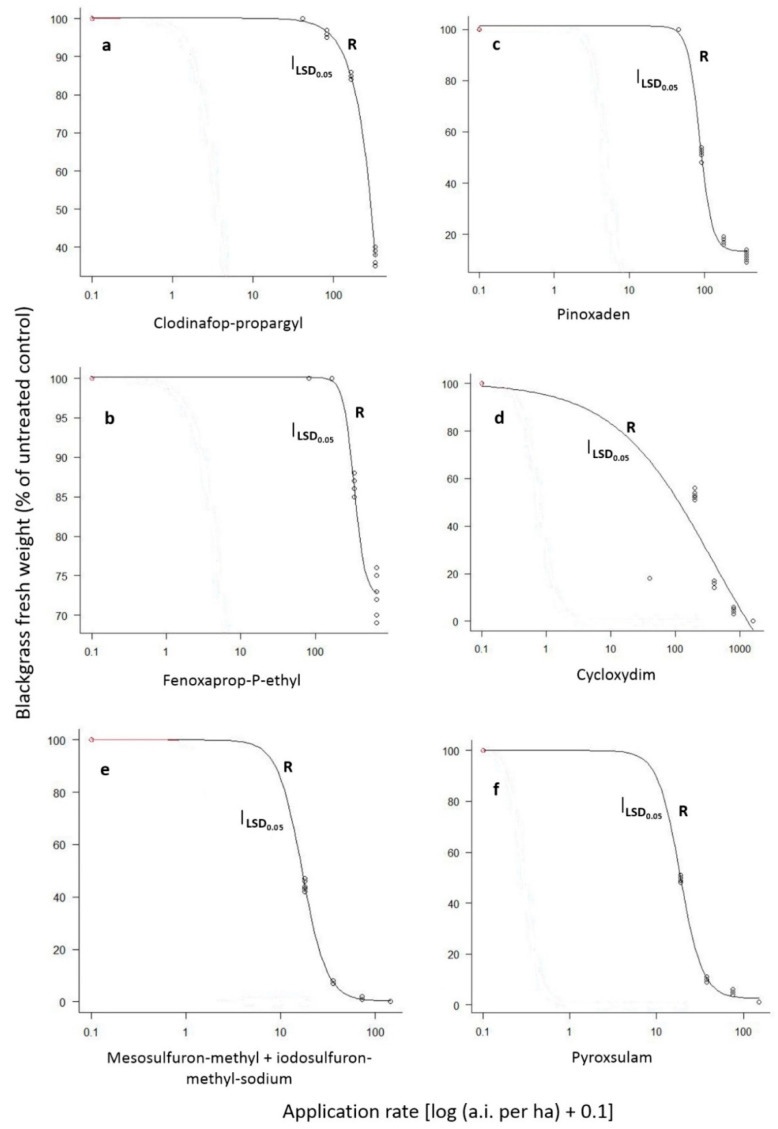
Fresh weight reduction (% of untreated control) of the R blackgrass population as affected by the ACCase-inhibiting post-emergence herbicides clodinafop-propargyl (**a**), fenoxaprop-P-ethyl (**b**), pinoxaden (**c**), or cycloxydim (**d**) and the ALS-inhibiting post-emergence herbicides mesosulfuron-methyl + iodosulfuron-methyl-sodium (**e**) or pyroxsulam (**f**). Values of each herbicide rate are means of eight replicates.

The ANOVAs performed separately for each blackgrass population indicated significant (*p* < 0.001) herbicide, rate and herbicide × rate interaction. Therefore, these interaction means are presented in Figure 3. More specifically, the application of the 1/8× and 1/4× rates of clodinafop-propargyl, fenoxaprop-P-ethyl, pinoxaden, or cycloxydim resulted in 74% and 96%, 61% and 87%, 75% and 99% or 100% and 100% control, respectively, of the S population. Similarly, the 1/8× rate of mesosulfuron-methyl + iodosulfuron-methyl-sodium or pyroxsulam resulted in 96% or 100% control, respectively, whereas all other rates provided excellent control (100%). In contrast, the application of the 1×, 2×, 4×, and 8× rates of clodinafop-propargyl or fenoxaprop-P-ethyl resulted in 0%, 4%, 15% and 62% or 0%, 0%, 14%, and 27% control of the R population, respectively, (Figure 3a,b). In addition, the application of pinoxaden at field label rates of 1×, 2×, 4×, and 8× resulted in 0%, 48%, 83%, and 89% fresh weight reduction, respectively, (Figure 3c). Furthermore, cycloxydim applied at 1×, 2×, 4×, and 8× rates provided 46%, 83%, 96%, and 100% control, respectively, (Figure 3d).

The application of 1×, 2×, 4×, and 8× rates of mesosulfuron-methyl + iodosulfuron-methyl-sodium provided 56%, 93%, 99%, and 100%, control of the R blackgrass population, respectively, (Figure 3e), while the respective control of pyroxsulam was 50% 90%, 95%, and 99% (Figure 3f). Although *GR*_50_ values are more suitable to compare the susceptibility between R and S populations, the *GR*_50_ values for the S population were not estimated as the lower rate of all herbicides (1/8×) resulting in more than 50% growth reduction. Regarding the R population, the estimated *GR*_50_ values for clodinafop-propargyl, fenoxaprop-P-ethyl, pinoxaden, cycloxydim, mesosulfuron-methyl + iodosulfuron-methyl-sodium or pyroxsulam were 1068.89, 332.21, 86.42, 400.08, 16.85, and 18.55 g ai ha^−1^ (Table 2), respectively, taking into consideration that their respective recommended rates are 40.8, 82.8, 45, 200, 15 + 3, and 18.75 g ai ha^−1^.

### 3.2. Rate-Response to Pre-Emergence Herbicides

The pre-emergence application of either prosulfocarb or the formulation of chlorotoluron + diflufenican, even at half their recommended field label rate, provided 97–98% fresh weight reduction in both R and S blackgrass populations.

### 3.3. Amplification and Sequencing of the ACCase Gene Fragment

**Figure 4:**
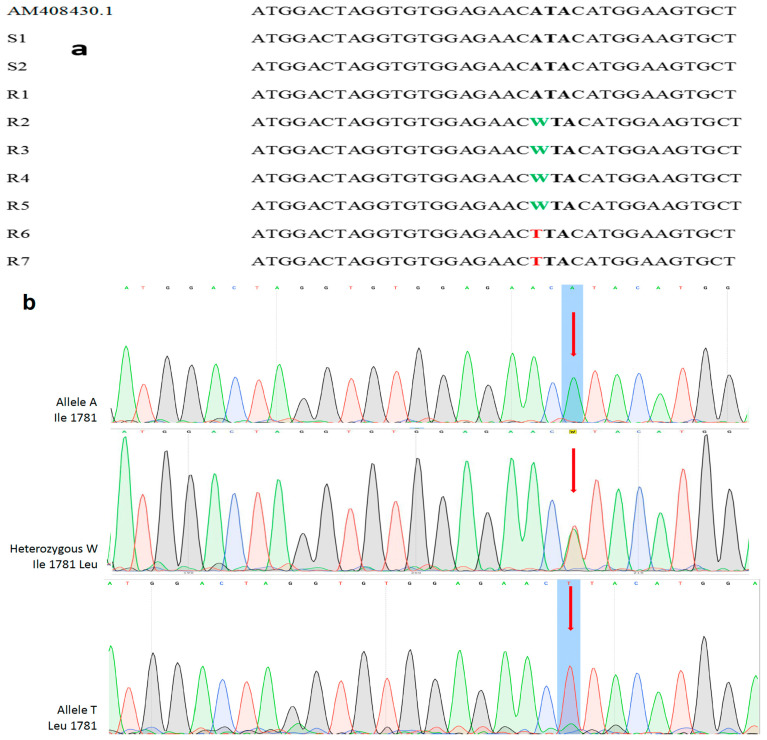
(**a**) Nucleotide sequence alignment of the *ACCase* gene taken from plants of the susceptible (S) and the ACCase R blackgrass population (first sample). The second and third samples represent the DNA sequences of the S population. The observed polymorphisms (point mutations) are highlighted in bold and correspond to the substitution of Ile1781 to Leu1781 position of the *ACCase* gene. IUPAC-IUB nucleotide codes: ATA (Il), TTA (Leu), WTA [TTA/ATA (Il/Leu)]. (**b**) Point mutations were detected in the first nucleotide in the codon 1781 (indicated by red arrows) at chromatograms in the analyzed blackgrass samples. IUPAC-IUB nucleotide codes: ATA (Il), WTA [TTA/ATA (Il/Leu)], TTA (Leu).

The alignment of the nucleotide sequences of the amplified *ACCase* gene fragments revealed that two out of seven sequenced R samples contained a single point homozygous mutation leading to the replacement of an Ile codon with a Leu codon at the 1781 site in the *ACCase* gene. Moreover, four out of seven sequenced R samples were heterozygous for the same mutation (Figure 4a). Finally, one sample of the R population (R1) and the two sequenced samples of the S population (S1, S2) were found with the wild-type Ile (ATA) genotype. Collectively, regarding the seven R samples molecularly examined, two were homozygous and four were heterozygous for the Ile/Leu alleles, whereas one sample of the R population (R1) and the two S samples were homozygous for the wild-type Ile genotype (Figure 4b). Furthermore, both S and R blackgrass populations harbored the wild-type allele in the remaining three genetic loci (Ile2041, Asp2078, Gly2096) that were investigated.

### 3.4. Metabolism Study

**Figure 5:**
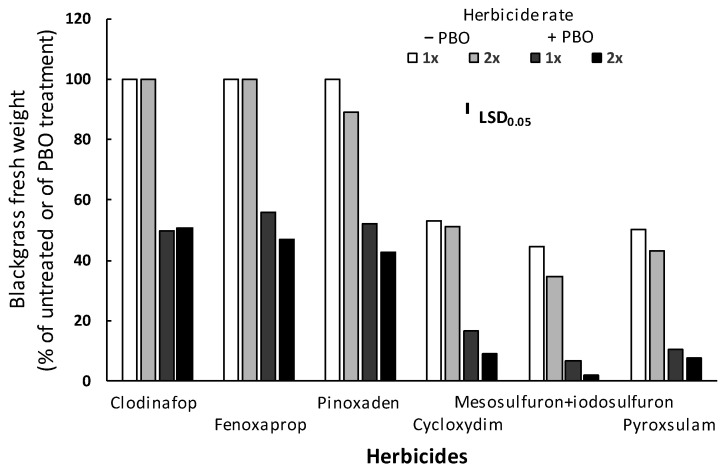
Effect of piperonyl butoxide (PBO) on the efficacy of the ACCase-inhibitors clodinafop-propargyl, fenoxaprop-P-ethyl, pinoxaden and cycloxydim, as well as of the ALS-inhibitors mesosulfuron-methyl + iodosulfuron-methyl-sodium and pyroxsulam against the aboveground fresh weight of the R blackgrass population. R populations were all homozygous in *ACCase* mutant as revealed by Sanger sequencing.

The ANOVA performed indicated significant (*p* < 0.001) main effects for herbicide, rate, and PBO, as well as a significant herbicide × rate × PBO interaction. Thus, these interaction means are presented in Figure 5. The fresh weight of the R blackgrass plants treated only with PBO did not differ from that of the untreated (control) plants, while the application of PBO two hours before the herbicide treatments considerably increased the herbicidal effect of ACCase- and ALS-inhibitors against the R blackgrass population. In detail, the pre-treatment of PBO followed by the application of clodinafop-propargyl, fenoxaprop-P-ethyl, pinoxaden, or cycloxydim at 1× and 2× label field rates resulted in increased fresh weight reduction of 50.1% and 49.5%, 44.0% and 53.0%, 47.9% and 52.3%, or 68.5% and 82.6%, respectively, compared with those of plants treated only by the two rates of the herbicides. Moreover, PBO pre-treatment followed by the 1× and 2× rates of the ALS-inhibitors mesosulfuron-methyl + iodosulfuron-methyl-sodium or pyroxsulam resulted in 84.8% and 94.8% or 79.1% and 82.0% fresh weight reduction, respectively, which was higher than the fresh weight reduction caused by the two rates of the herbicides only (Figure 5).

### 3.5. Blackgrass Competition with Winter Wheat

**Figure 6:**
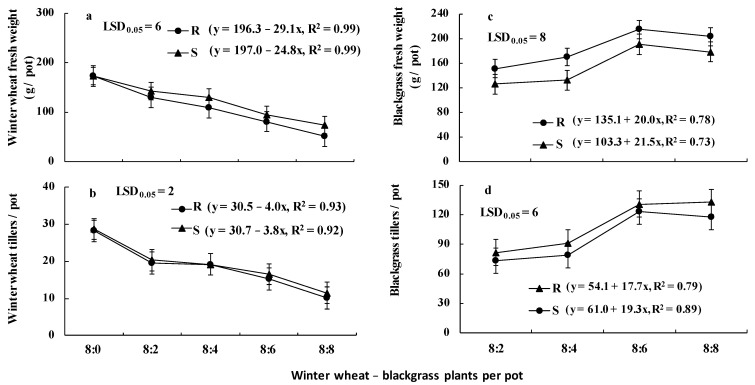
Fresh weight (**a**) and tiller number (**b**) of winter wheat (cultivar Zanzibar) grown free from the presence and in competitive interaction with the R and the S blackgrass populations, as well as fresh weight (**c**) and tiller number (**d**) of blackgrass [mean of eight replicates (pots)]. R populations were all homozygous in the *ACCase* mutant as revealed by Sanger sequencing.

The ANOVA performed for the winter wheat data indicated that there was no repetition time × treatment interaction. However, the tiller number was affected by the winter wheat–blackgrass ratio (*p* < 0.001), while fresh weight was affected (*p* < 0.001) by blackgrass population, winter wheat–blackgrass ratio, and blackgrass population × winter wheat–blackgrass ratio interaction. Similarly, both the tiller number and fresh weight of blackgrass were affected (*p* < 0.001) by blackgrass population, winter wheat–blackgrass ratio, and blackgrass population × winter wheat–blackgrass ratio interaction. So, the blackgrass population × winter wheat–blackgrass ratio interaction means are presented in Figure 6. More specifically, the fresh weight (Figure 6a) and tiller number (Figure 6b) produced by winter wheat plants significantly decreased with increasing density of the R and S blackgrass populations, whereas significant differences between populations were observed only in fresh weight. The presence of 2, 4, 6, and 8 R plants co-existing with eight winter wheat plants resulted in 24.6%, 36.8%, 53.1%, and 69.9% reduction in the fresh weight of wheat plants, respectively, (Figure 6a). The corresponding decrease by the competition of the S blackgrass plants was 17.2%, 24.8%, 45.4%, and 57.6%, respectively, (Figure 6a). Regarding the fitted linear regression equations, the slope of wheat fresh weight reduction due to R population was greater (29.1) compared to that of the S population (24.8) (Figure 6a).

Concerning the weed growth, the plants of the R blackgrass population produced 20%, 28%, 13% or 14% more fresh weight (Figure 6c) and 11%, 16%, 6%, or 13% more tillers (Figure 6d) than the S population, when 2, 4, 6, or 8 weed plants were grown in competitive interaction with 8 winter wheat plants, respectively. However, in contrast with winter wheat, the growth slopes of the S population were slightly greater in comparison with the R population (Figure 6c,d).

## 4. Discussion

### 4.1. Whole-Plant Rate-Response to Post-Emergence Herbicides

The results of the present study indicated that the reduced control reported by a grower in a winter wheat field in the county of Grevena (northwestern Greece) was due to the evolution of cross-resistance to ACCase-inhibiting herbicides. In addition, the revealed low efficacy for the label recommended rate of the ALS-inhibitors mesosulfuron-methyl + iodosulfuron-methyl-sodium or pyroxsulam against the R population strongly supports field-selection for multiple resistance. These findings could result from the repeated use of clodinafop propargyl and fenoxaprop-P-ethyl in dryland cereal production systems. Probably, the two selective aryloxyphenoxypropioanate herbicides acted as selecting agents of field-evolved ACCase-herbicide resistance, while the reduced efficacy of the ALS-inhibitors limits their choice as effective alternatives for selective grass weed control in winter cereals. Similarly, Lan et al. [29] found in China winter wheat fields two blackgrass populations with cross-resistance to clodinafop-propargyl and fenoxaprop-P-ethyl and simultaneously multiple-resistance to mesosulfuron-methyl and pyroxsulam. Although resistance factor (*GR*_50_ of the R/*GR*_50_ of the S) values were not calculated for the herbicides tested as the lower rate of all herbicides (1/8×) resulting in more than 50% growth reduction in the S population, the comparison of the *GR*_50_ of the R/recommended rate ratios of each herbicide indicated that 26.2, 4.01, 1.92, 2.0, 0.94, and 0.98 times higher than the recommended rate of clodinafop propargyl, fenoxaprop-P-ethyl, pinoxaden, cycloxydim, mesosulfuron-methyl + iodosulfuron-methyl-sodium, and pyroxsulam, respectively, were needed to reduce the fresh weight of the R population by 50%.

### 4.2. Rate-Response to Pre-Emergence Herbicides

The excellent efficacy of the soil-applied residual herbicide prosulfocarb or the formulation of chlorotoluron + diflufenican against the R and S blackgrass populations is in conformity with the findings of Baily et al. [30], which indicated that prosulfocarb and chlorotoluron + diflufenican can be used as a very effective alternative chemical option to either delay the selection for resistance to post-applied ACCase- and ALS-inhibiting herbicides or manage already field-evolved resistant blackgrass populations in small cereal arable fields. In addition, long-lasting residual herbicide mixtures with different sites of action (such as the formulation of chlorotoluron + diflufenican) could be very useful in achieving robust control of blackgrass emergence in crop fields with no appreciable high risk in terms of selective pressure and subsequent spread of field-selected resistance [30]. Regarding soil applied herbicides, many European blackgrass populations infesting arable fields display low-level resistance to flufenacet and other soil-applied herbicides (pendimethalin, prosulfocarb, S-metolachlor, pethoxamid), and the differences in their sensitivity are correlated with enhanced herbicide metabolism, catalyzed by glutathione S-transferases [31].

### 4.3. Amplification and Sequencing of the ACCase Gene Fragment

The molecular study revealed that the underlying mechanism of resistance was target-site mediated. The molecular analysis of the *ACCase* gene fragment of the R blackgrass population proved that a single nucleotide polymorphism in the CT domain of the homomeric ACCase resulted in amino acid substitution at position 1781. This enzyme sequence modification imparted resistance to ACCase inhibitors and was therefore responsible for the phenotypic response determined in the whole-plant rate-response assays. The fact that two of the sampled R plants were homozygous and four were heterozygous for the Ile/Leu alleles of *ACCase* gene strongly suggests that cross-resistance has been evolved recently. However, the homozygous R1 plant with the wild-type Ile genotype could be attributed to the NTSR mechanism confirmed in our metabolism study, while the two homozygous S samples for the wild-type Ile genotype supports our choice for the selection of this population as S.

The detected Ile1781Leu mutation conferring ACCase-inhibitor resistance is the most frequently identified in ACCase-resistant blackgrass and other important grass species, and, according to Jang et al. [32], the grass weed populations carrying the Ile1781Leu mutation exhibit broad cross-resistance to the three chemically dissimilar classes of ACCase inhibitors (aryloxyphenoxypropionates, cyclohexanediones, and phenylpyrazolin). The Ile1781 residue, being a part of the target protein binding site, is located in a binding pocket occupied by a methyl or ethyl group in all chemically dissimilar ACCase-inhibiting herbicides [33], but the replacement of Ile1781 with Leu or Val in the *ACCase* gene is responsible for relatively low resistance indexes [32]. As stated by Kaundun [14], it is difficult to overcome resistance to ACCase inhibitors in arable fields, after it has been selected for and established, despite subsequent applications of different herbicide modes of action against the field-selected resistant populations. More specifically, implementation of alternative, non-chemical weed control measures such as seedbed sanitation, the timely alteration of sowing dates and increased sowing densities, direct sowing, physical control methods, field hygiene methods and regular monitoring [34], although crucial for managing the blackgrass seed bank, have a small contribution to the overall percentage of resistant individuals in the field-evolved resistant populations. Importantly, the *ALS* gene of the R population was not sequenced and aligned because most ALS-inhibitor target-site resistant, field-selected populations in blackgrass and other weed species display very high resistance indexes [7,16,19]. In contrast, in the present study control of the R blackgrass population was compromised only at the recommended label rate of ALS-inhibiting herbicides, whereas their higher rates provided good to excellent control.

### 4.4. Metabolism Study

The significant increase in control determined after the combined application of post-emergence herbicides (of both modes of action) with PBO (applied 2 h before herbicide application), coupled with the high levels of resistance assessed, strongly suggests that the evolved resistance also involves a non-target-site-based resistance (NTSR) mechanism based on increased herbicide degradation into non-toxic entities by either cytochrome P450 or glutathione transferases (GST) [35] in the R blackgrass population studied. The NTSR is recognized as the predominant resistance mechanism impacting dramatically on the efficacy of ACCase-inhibiting herbicides [17,36], and regarding France, a large-scale blackgrass survey revealed that most ACCase-inhibitor-resistant blackgrass plants did not contain a target-site mutation conferring herbicide resistance [15,37]. Although NTSR affects dissimilar chemistries and herbicide modes of action, it is compound-specific within and between ACCase inhibitors [14,18]. This is confirmed by the recent results reported by Tian et al. [38], who found that overexpression of cytochrome P450 CYP71AF43 contributes resistance to fenoxaprop-P-ethyl in blackgrass and cross resistance to pinoxaden and clethodim, whereas CytP450 (CYP86B1 and CYP71C1), GST (GSTT1) and glycocyl transferase (UGTZ3C, CGT, and especially GT8) genes are responsible for decreased sensitivity to fenoxaprop-P-ethyl and various ACCase-inhibitors (haloxyfop-P-ehtyl, quizalofop-P-ethyl, clodinafop-propargyl, sethoxydim, clethodim, and pinoxaden).

### 4.5. Blackgrass Competition with Winter Wheat

The present study conducted to detect and assess possible differences in the competitive response of R and S blackgrass populations against winter wheat revealed that wheat suffered a greater fresh weight reduction when grown in competitive interaction with the R blackgrass population than the S population. Therefore, findings of the present study suggest that the R population is characterized by a higher competitive ability against wheat compared to the S population. This could be attributed to the fact that the R blackgrass population produced more fresh weight and tillers than the S population, suggesting a fitness advantage for the R population due to ACCase target-site and enhanced herbicide metabolism. These findings are in accordance with those of Wang et al. [39], who reported a higher fitness of the 1781Leu mutant allele in ACCase-herbicide-resistant green foxtail [*Setaria viridis* (L.) P. Beauv.], compared to the wild type. Délye et al. [40], who studied germination dynamics and seedling emergence of wild and mutant blackgrass genotypes, found that the Gly2078 ACCase mutant isoform caused accelerated germination, whereas the most commonly field-evolved Leu1781 mutant isoform was responsible for a delayed germination of blackgrass. In contrast to these findings, Menchari et al. [41] reported that the heterozygous and homozygous subpopulations arising from the same parental blackgrass population, carrying the 1781 and 2041 mutant alleles, suffered no fitness cost compared to the wild-type allele identified in the S population. Also, the gene mutation leading to the ACCase amino acid substitution Ile1781Leu showed no pleiotropic effects on ACCase activity and kinetics and was responsible for a negligible impact on various fitness-related growth traits in rigid ryegrass (*Lolium rigidum* Gaudin.) [42,43] and in smooth barley (*Hordeum glaucum* Steud.) [44]. Lastly, according to Darmency et al. [45] and Kaundun [14], the fitness cost associated with the above-mentioned ACCase mutant allele depends on the population genetic background and field environment, while Colbach et al. [46] reported that the frequency and prevalence of mutant alleles conferring target-site mediated ACCase resistance are influenced mainly by their associated fitness penalty or benefit rather than by the number and rate of herbicide applications or the cultural practices employed to manage ACCase resistance.

Concerning the effect of non-target-site mediated ACCase-inhibitor resistance (NTSR) on the growth traits of the blackgrass population, the results reported by Keshtkar et al. [47,48] indicated a reduced emergence of buried seeds, although they produced similar vegetative aboveground biomass and reproductive fitness under competition with winter wheat. Moreover, Comont et al. [23] found that a NTSR blackgrass population displayed pleiotropic impacts on plant growth, development, and resource partitioning, but they concluded that resistance was not associated with a consistent reproductive fitness penalty. On the contrary, Vila-Aiub et al. [24] reported an ecological fitness cost and weaker competitive response of NTSR rigid ryegrass populations due to enhanced metabolism against wheat plants.

## 5. Conclusions

The present study confirmed the evolution of high-level ACCase (clodinafop-propargyl, fenoxaprop-P-ethyl, pinoxaden, and cycloxydim) and a medium-level of ALS-inhibitor (mesosulfuron-methyl + iodosulfuron-methyl-sodium and pyroxsulam) multiple-resistance in a blackgrass population selected from a wheat monoculture field. The resistance was target-site mediated, due to a point mutation that resulted in the substitution of Ile by Leu in position 1781 of the ACCase gene, and the level of resistance was enhanced by a co-existing metabolism due possibly to CYP450. The 1781Leu ACCase mutant allele conferring resistance slightly increased blackgrass growth, which was confirmed by its greater competitive ability against winter wheat as compared with that of the S population. Lastly, the R and S blackgrass populations were most effectively controlled by the registered pre-emergence applied herbicides prosulfocarb or the formulation of chlorotoluron + diflufenican in winter wheat, suggesting these herbicides as alternative chemical options for the control of blackgrass and other grass weeds.

## Figures and Tables

**Table 1:** Primers used for ACCase target-site mutations.

Primer Pair	Sequences	Targeting Mutation	Tm	Reference
ACcp1	5′-CAACTCTGGTGCTIGGATIGGCA 3′-5′-GAACATAICTGAGCCACCTIAATATATT-3′	Ile-1781	56	[23]
Alo_acc2041	5′-GCAAAGAGATCTTTTTGAAGGA-3′ 5′-GGATCAAGCCTACCCATGCA-3′	Ile-2041	54	[24]
Alo_acc2078	5′-GTGGAGGAGCCTGGGTCGTGATT-3′ 5′-GGATCAAGCCTACCCATGCA-3′	Asp-2078	54	[24]
Alo_acc2096	5′-GCTATGCTGAGAGGACTGCAAAG-3′ 5′-GGATCAAGCCTACCCATGCA-3′	Gly-2096	54	[24]

**Table 2:** Parameters of the four-parameter log-logistic curves describing the relationship between clodinafop-propargyl, fenoxaprop-P-ethyl, pinoxaden, cycloxydim, mesosulfuron-methyl + iodosulfuron-methyl-sodium, or pyroxsulam application rate and fresh weight of the R blackgrass population.

Blackgrass Resistant Population						
	Clodinafop	Fenoxaprop	Pinoxaden	Cycloxydim	Mesosulfuron + Iodosulfuron	Pyroxsulam
**Parameters**			(±SE)			
B	2.13 ± 0.08 ***	5.99 ± 1.33 ***	5.33 ± 0.48 ***	0.56 ± 0.24 *	3.37 ± 0.09 ***	3.39 ± 0.13 ***
C	−739.91 ± 572.54 ns	72.43 ± 0.62 ***	13.40 ± 0.63 ***	−51.43 ± 46.52 ns	0.19 ± 0.27 ns	2.50 ± 0.40 ***
D	100.16 ± 0.26 ***	100.14 ± 0.28 ***	101.26 ± 0.66 ***	100.36 ± 5.64 ***	100.00 ± 0.33 ***	100.01 ± 049 ***
***GR*_50_ (g ai ha^−1^) **	1068.89 ± 109.41 *	332.21 ± 4.48 ***	86.42 ± 0.67 ***	**400.08 ± 430.52 ns**	16.85 ± 0.08 ***	18.55 ± 0.13 ***
Lower	238.57	323.13	85.06	−473.06	16.68	18.28
Upper	1899.22	341.29	87.77	1273.23	17.02	18.81

ns, * and ***: not significant, significant at 0.05 and 0.001, respectively. *GR*_50_ values are in bold.

## Data Availability

The original contributions presented in the study are included in the article, further inquiries can be directed to the corresponding authors.
